# Microstructural Tailoring and Enhancement in Compressive Properties of Additive Manufactured Ti-6Al-4V Alloy through Heat Treatment

**DOI:** 10.3390/ma14195524

**Published:** 2021-09-24

**Authors:** Byungmin Ahn

**Affiliations:** 1 Department of Materials Science and Engineering, Ajou University, Suwon 16499, Korea; byungmin@ajou.ac.kr; Tel.: +82-31-219-3531; Fax: +82-31-219-1613; 2 Department of Energy Systems Research, Ajou University, Suwon 16499, Korea

**Keywords:** additive manufacturing, powder metallurgy, annealing, implants, biomaterials, titanium, selective laser melting

## Abstract

Among laser additive manufacturing, selective laser melting (SLM) is one of the most popular methods to produce 3D printing products. The SLM process creates a product by selectively dissolving a layer of powder. However, due to the layerwise printing of metal powders, the initial microstructure is fully acicular α′-martensitic, and mechanical properties of the resultant product are often compromised. In this study, Ti-6Al-4V alloy was prepared using SLM method. The effect of heat treatment was carried out on as-built SLM Ti-6Al-4V alloy from 650–1000 °C to study respective changes in the morphology of α/α′-martensite and mechanical properties. The phase transition temperature was also analyzed through differential thermal analysis (DTA), and the microstructural studies were undertaken by optical microscopy (OM) and scanning electron microscopy (SEM). The mechanical properties were assessed by microhardness and compressive tests before and after heat treatment. The results showed that heat treated samples resulted in a reduction in interior defects and pores and turned the morphology of the α′-martensite into a lamellar (α + β) structure. The strength was significantly reduced after heat treatment, but the elongation was improved due to the reduction in columnar α′-martensite phase. An optimum set of strength and elongation was found at 900 °C.

## 1. Introduction

SLM additive manufacturing technology produces parts by stacking powders layer by layer from a metal powder bed [1]. The immediate fusion of powder particles provides superior efficiency in large sized and complex manufacturing of products. The molten powder experiences rapid solidification of the order of 103–106 °C/s that produces a novel microstructure of Ti-6Al-4V alloys. To date, SLM has been extensively applied in the manufacturing of near net shape design and high-performance products, representative aircraft parts, and medical and dental implants, etc. The SLM process is advantageous due to its economic benefits such as low wastes, faster processing times, and the ability to manufacture almost any shape [2,3]. There is no fear of a reduction in precision due to tool wear and a large number of samples can be produced in the same batch manufacturing with the possibility of a fully automatic process. Due to the difference in shapes and sizes of human body tissues and organs of each person, 3D printing is highly utilized in the medical field and future designs of personalized implants seem to be growing very fast in medical technology [4,5]. The range of application can be greatly expanded owing to its CAD-based design approach for the customized implants and prostheses for individual patients [3]. The most widely studied and used alloy in the above field is Ti-6Al-4V alloy as, among them, it has superior strength, toughness, and corrosion resistance [6].

Ti-6Al-4V alloy is a typical duplex phase alloy, and the rapid heating of the selective laser melting method and the quenching cycle above the critical temperature make the initial microstructure of the part consist only of the acicular α′-martensite phase (here the critical temperature is the temperature at which α′-martensite is formed when quenched at that temperature) [7]. The formation of α′-martensite results in an anisotropy of the material which affects the ductility of the alloy. Traditional Ti-6Al-4V alloy exhibits two different types of microstructures depending upon the morphology of α phase. It can be either lamellar or equiaxed (globular) [8,9,10]. The mechanical properties are characterized due to the mixture of these types of microstructures, such as bimodal [9,10], tri-modal [10,11], or a mixture of coarse and fine lamellae [10]. However, in SLM manufactured Ti-6Al-4V alloys, the metal powder is heated up and quickly melted by a laser beam owing to a high temperature gradient (10^6^–10^8^ K/s) [12]. Such extreme solidification rates BCC-β phase transforms into metastable HCP-α′ martensite. The final microstructure is highly anisotropic with columnar grains and results in loss of plasticity [13]. Therefore, the Ti-6Al-4V alloy produced by the SLM generally shows high yield strength but is not suitable for application to various products due to its very limited ductility and low fatigue resistance [14,15]. Additionally, the local laser melting, layered manufacturing results in the columnar grains and other printing defects such as build porosity, residual stresses, segregated or unmelted powders [16]. These residual stresses and anisotropies deteriorate mechanical properties and should be avoided as they may cause local distortion and microcrack formation [17,18,19].

Vrancken et al. performed heat treatment of SLM produced Ti-6Al-4V alloy at 850 °C for 2 h. They found a significant improvement in ductility by 13% over as-fabricated Ti-6Al-4V alloy. In their results, the martensite α′ phase was transformed to a lamellar α + β structure according to the annealing condition below the β-transus [20]. Kim et al. also performed heat treatment on SLM manufactured Ti-6Al-4V alloy. Their results indicated a decline in the hardness and yield strength value of SLM Ti-6Al-4V after heat treatment, but the mechanical anisotropy disappeared [21]. Similarly, Wu et al. [22] established the relationship between heat treatment and hardness in a systematic way. Their study indicated a double peak phenomenon in measured Vickers microhardness due to the decomposition and reformation of martensite at 500 and 1000 °C. As such, heat treatment enables the release of residual stress and control mechanical properties through effective microstructure control. The heat-treatment of SLMed Ti-6Al-4V is generally carried out to improve the microstructural properties. However, despite several studies made in the past, the optimal set of heat treatment conditions for meeting the best hardness and compression strength are not consistent in the literature owing to their complex thermal history. Conditions for the adjustment of typical α + β lamellar microstructure contributes to an improvement in ductility as shown in the past [23], a structure aimed in the present study.

This study investigated the phase change and mechanical performance of Ti-6Al-4V alloy manufactured with SLM and followed by heat treatment study. The microstructures of the SLM Ti-6Al-4V were analyzed in as-built and after heat treatment of specimens. The microhardness and compressive properties of the samples were also studied with respect to the heat treatment temperatures.

## 2. Materials and Methods

### 2.1. Materials

Ti-6Al-4V ELI (Extra Low Interstitial) powder (EOS GmbH, Krailling, Germany) produced through the gas atomization method was used as the basic powder material for SLM. The powder particles had a diameter of 15–58 µm.

### 2.2. Selective Laser Melting

All SLM parts were produced under the EOS M290 Default Laser Exposure condition on the EOS M290 metal 3D printer, Krailling, Germany. The specimen size after printing was 30 mm × 13 mm × 5 mm as-fabricated along the XY direction with laser power 125 W and a scan speed of 480 mm/s.

### 2.3. Heat Treatment

The heat treatment of as-built Ti-4Al-4V samples was performed in a tube furnace, (HORIBA Advanced Techno, Co., Kyoto, Japan) at a heating rate of 10 °C/min in an N2 atmosphere. The heat treatment was performed under five conditions of 650 °C, 750 °C, 800 °C, 900 °C, and 1000 °C for 2 h. The cooling cycle proceeded at an average cooling rate of 0.04 °C/s.

### 2.4. Microstructural Investigations

Microstructure analysis was carried out by grinding the sample to a fine size up to 2000 Grit with SiC grinding paper, polishing 1 μm with poly diamond suspension, and fine polishing to 0.02 µm using Vibromet equipment—Buehler, Tokyo, Japan. The microstructure of the as-built and post heat-treated specimens were observed by optical microscope (OLYMPUS BX51RF, Tokyo, Japan), scanning electron microscope (JEOL SEM JSM 7200F, Tokyo, Japan) coupled with an energy dispersive X-ray detector for compositional analysis. Additionally, the phase evolution of the SLM Ti-6Al-4V samples was confirmed through X-ray diffraction analysis (XRD, Brucker’s D8 Advance, Karlsruhe, Germany). XRD was performed at 0.5°/min in the range of 20–80°, and the scanning step was 0.01°. To visualize the microstructural features in detail in OM studies, the polished specimens were rinsed by Krolls etchant, ES Laboratory, Glendora, CA, USA (92 mL of H_2_O, 6 mL of HNO_3_, and 2 mL of HF) in the direction perpendicular to the surface.

### 2.5. Mechanical Properties

The microhardness of different samples was determined by Vicker’s hardness tester (Mitutoyo HM-200, Janagawa, Japan). The measurements were carried out at a load of 200 g for 10 s. Further, the compressive tests were performed on the as-built and heat-treated Ti-6Al-4V specimens by micromachining the samples in dimensions of 3 mm × 3 mm × 6 mm. The compression test was carried out at a strain rate of 10^−3^/s along the perpendicular build direction. The elongation and strength values were extracted from the compressive stress–strain diagrams. Further, the absorption energy up to fracture was determined from the integrated area of the stress–strain diagrams and reported. A total of 5 specimens were tested and average values were reported.

## 3. Results and Discussions

### 3.1. Phase Transformation Studies

**Figure 1:**
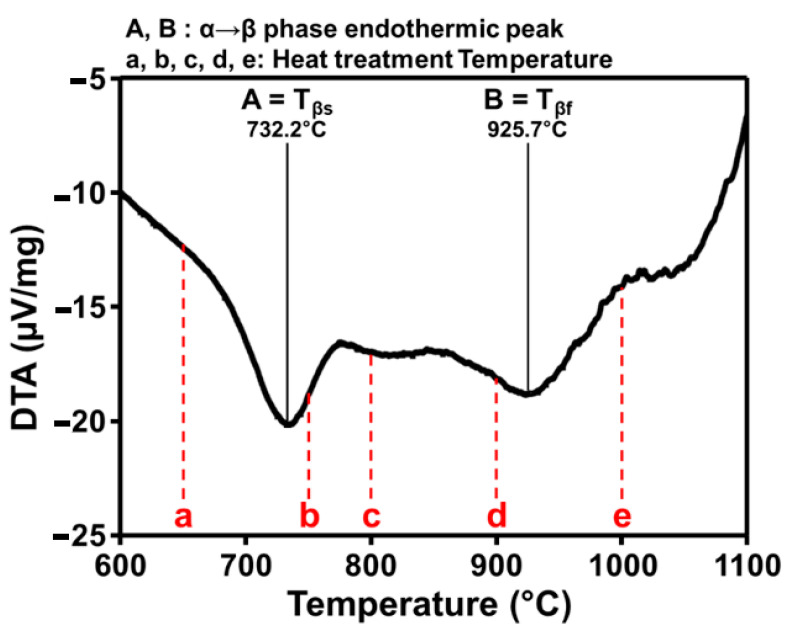
DTA thermogram of the SLM Ti-6Al-4V alloy.

The DTA traces of the samples are shown in Figure 1. A phase transition point was found near 730 °C before the β-transus temperature and, therefore, the heat treatment was performed at five different temperatures starting from the temperature corresponding to the two-phases (α + β) region.

Points A and B indicate the endothermic peaks of the corresponding α and β phases. Before 650 °C, only α-phase was prominent, while β phase formed later beyond heat treatment at 750 °C. The β-transus temperature of SLM Ti-6Al-4V sample is 985.81 °C as reported by Yolton et al. [24]. In the present study, the β-transus temperature was slightly lower value than the 925.7 °C. Therefore, in this study, we defined the heat treatment temperatures across the temperature regions within 800 °C, 900 °C, and β-transus, or higher temperature around 1000 °C.

### 3.2. Phase Evolution

**Figure 2:**
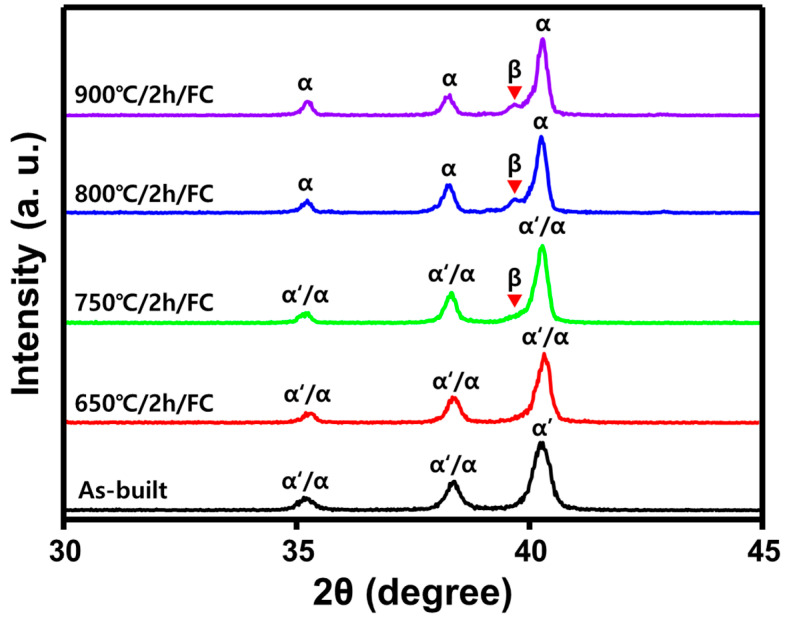
XRD patterns of as-built and heat-treated SLM Ti-6Al-4V at various temperatures.

Figure 2 shows the XRD patterns of as-built Ti-6Al-4V and heat-treated Ti-6Al-4V samples. In this study, only peaks between 30–45° were compared and analyzed in order to compare only the peaks of the α-phase, α′-phase, and β-phase that need to be controlled.

In the as-built alloy, the presence of α or α′-martensite phases were noticed. An acicular martensite phase is often present in Ti-6Al-4V alloys with a fewer concentration of V (β-stabilizer). The hexagonal close-packed structure is distorted during rapid solidification and α to α′ transformation occurs. Therefore, the as-built specimen shows a fully martensitic structure due to a fast-cooling rate in SLM processing. On the other hand, in heat-treated specimens after 750 °C, the β-phase is noticed which continues to evolve at higher heat treatment conditions until 900 °C. The β-peak shifts to a lower 2θ near 37°, showing decomposition of α′ to α-phase [2,3].

### 3.3. Microstructure

**Figure 3:**
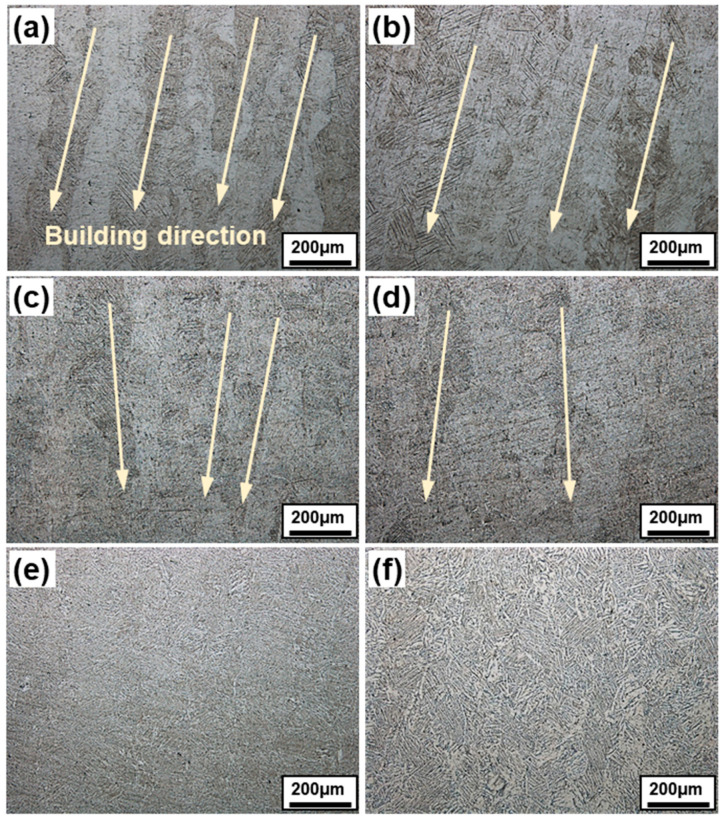
Optical micrographs of SLM Ti-6Al-4V alloy in as-built and heat-treated conditions. (**a**) As-built, (**b**) 650 °C, (**c**) 750 °C, (**d**) 800 °C, (**e**) 900 °C, and (**f**) 1000 °C.

Figure 3 displays OM images of as-built and heat treated SLM Ti-6Al-4V alloys. It can be seen that a coarse columnar grain growth occurs (100 µm in width) in the build direction, which is a characteristic of SLM manufacturing process. These elongated columnar grains are formed by heat flow in a single direction during solidification. Compared to cast counterparts, the heat flow path has no specific directionality. However, in laser additive manufacturing, due to a layerwise growth process, the columnar growth occurs in an epitaxial buildup of the partially remelted top surface of the previous solidified surface [19,21,22]. In this way, certain anisotropic directions are formed as prior-β grains, and this directionality adversely affects mechanical properties, except in very few cases.

The columnar grains were not as prominent in the heat-treated specimens compared to the as-built specimen (Figure 3a). Additionally, defects were also minimized in heat-treated samples (Figure 3b,c). It was confirmed that the anisotropy of the material was gradually removed as the heat treatment proceeded. This is clearer from the optical images shown in Figure 3d–f. At 900 °C, all the columnar grains became smaller in size. As the recrystallization temperature of the Ti-6Al-4V alloy is about 850 °C, it is judged that recrystallization occurs and all anisotropy is removed at 900 °C. Some α-grains gradually turned globular and overall anisotropy tended to disappear at this condition. A similar behavior is obtained by Zhang et al. while studying post-heat treatment effect on additive manufactured Ti-6Al-4V alloy [15].

**Figure 4:**
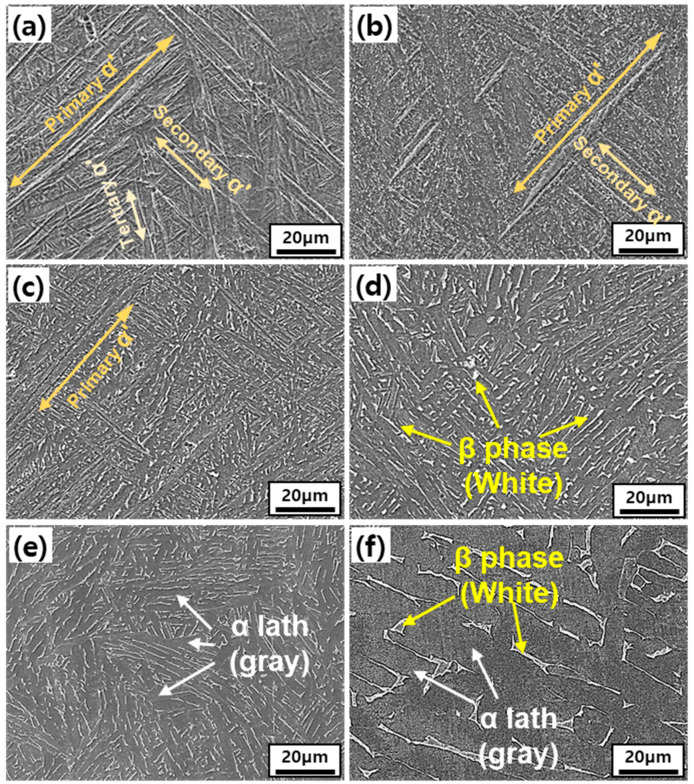
SEM images of as-built and heat-treated SLM Ti-6Al-4V. (**a**) As-built, (**b**) 650 °C, (**c**) 750 °C, (**d**) 800 °C, (**e**) 900 °C, and (**f**) 1000 °C.

Meanwhile, it is easier to understand the effect of heat treatment on grain size if we look the high resolution images of the interior of the columnar grains. Figure 4 shows the SEM images of the Ti-6Al-4V alloy in as-built and heat-treated conditions. There was a huge difference in the morphology of α-phase, as shown in Figure 4. The as-built specimen consisted of acicular α′-phase as also detected by XRD analysis. After heat treatment, α and β have co-existed in a basket weave type network [15]. The α′-martensite phase begins to decompose at 400 °C into α + β phases in a small amount after heat treatment at 650 °C (Figure 4b). After raising the heat treatment temperature to 750 °C, a fine β-phase was formed.

At 800 °C, α′-martensite had mostly disappeared and α + β lamellar structure began to dominate (Figure 4c). At 900 °C, only α + β lamellar structures exist. As the heat treatment temperature increases, the width between the lamellar structures increases but showed a similar tendency up to 900 °C. The lamellar structure has a different growth direction from the adjacent α lath, and the grain boundary cannot move in the axial direction of the α lath, which limits the growth (Figure 4d,e). However, when heat treatment was performed at 900 °C or higher, the element-partitioning effect became apparent, as all α′-martensite phases were decomposed and the diffusion rate enhanced due to the high temperature. It was confirmed that the lamella width at 1000 °C increased about 2.5 times (2.2 μm vs. 5.7 μm) compared to the lamella width at 900 °C (Figure 4f). The β phase was located at the boundary of the dense α-phase colony. These findings indicate that the defects and pores are reduced after heat treatment.

In addition, it is known that the martensite formation involves enhancement in twin and dislocation density of SLM Ti-6Al-4V which is higher than that of cast Ti-6Al-4V alloy [25,26]. Thus, the characteristic size on SLM α′-martensite is known to be higher than that of Ti-6Al-4V alloy. It is smaller than the α′-martensite phase of Ti-6Al-4V alloy. These dislocations and twins are believed to slow down the grain growth to form a fine α + β lamellar structure [15]. Therefore, it is important to determine the optimum heat treatment temperature for maintaining this fine lamellar structure.

### 3.4. Microhardness

**Figure 5:**
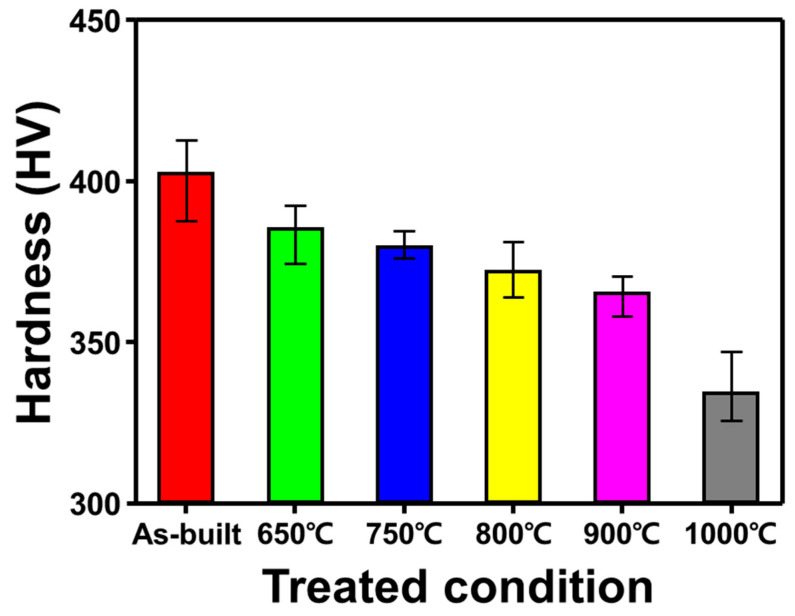
Micro Vickers hardness of SLM Ti-6Al-4V specimens heat-treated at various temperatures.

Figure 5 shows the change in the microhardness of SLM Ti-6Al-4V after heat treatment. It is observed that the microhardness of SLM Ti-6Al-4V alloy is correlated to its microstructure. The hardness of the as-built SLM Ti-6Al-4V sample was about 400 HV which deteriorated after heat treatment. Among the heat-treated samples, the sample heat-treated at 650 °C had the highest hardness (387 HV). The hardness of specimens changed slightly to 385 HV at 750 °C.

The reason the heat treatment conditions at 650 °C and 750 °C produce higher hardness than other conditions is due to a sufficient fraction of α′-martensite. The α′-phase begins to decompose to lamellar α + β structure at 800 °C. Consequently, the microhardness of specimens drop significantly. The microhardness of the SLM Ti-6Al-4V samples heat-treated at 1000 °C showed a lowest hardness value of 325 HV.

High-temperature heat treatment causes the elimination of internal stresses and coarsening of the α + β lamellar structure. Overall, the microhardness of SLM Ti6-Al-4V alloy depends on the shape, size, and fraction of the constituent phases. The decomposition of α′-martensite enhances the formation of α + β lamellar structure, the width of the lamellae increased at a higher heat treatment temperature [20]. Therefore, a significantly lower hardness value is observed at 1000 °C where the lamella width was higher (5.7 µm) as compared to that at 900 °C (2.2 µm) as already proven from the SEM investigations.

### 3.5. Compressive Test

**Figure 6:**
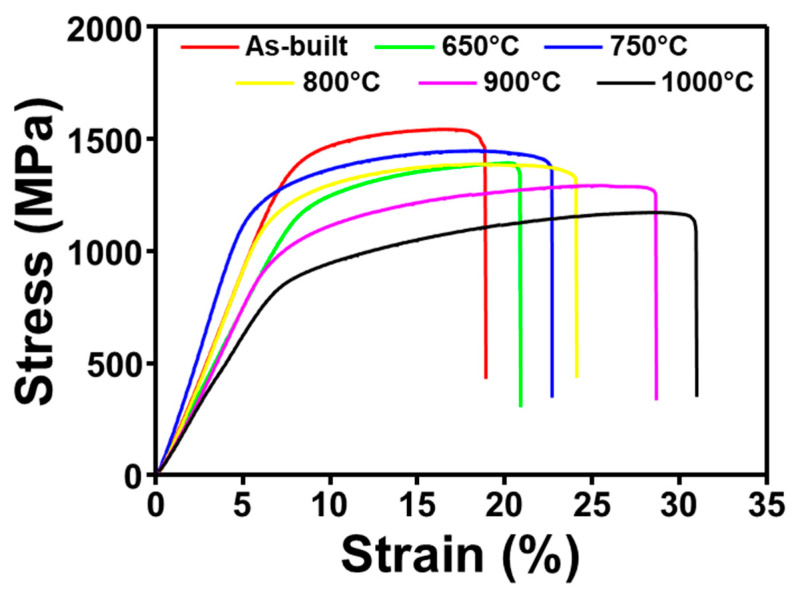
Compressive stress–strain diagrams of as-built and heat-treated SLM Ti-6Al-4V alloys.

Figure 6 shows the results of compressive tests conducted on the different samples to analyze their strength and elongation properties. We can see that the stress–strain diagrams show a remarkable change in the deformation region. The as-built sample showed higher deformability which decreased down with the heat treatment temperatures. It is already shown that the strength and elongation of the alloy are also closely related to the microstructure changed after heat treatment. The four important factors that affect the deformability of the as-built SLMed Ti-6Al-4V alloys are as follows [14,15].

(1) As-built sample is fully α′-martensite, which is brittle, a major cause of the inferior deformability of as-built SLM Ti-6Al-4V alloy.
(2) Morphological of α′-martensite is acicular which induces anisotropy and hence poor mechanical properties.
(3) The internal stresses in the sample due to a rapid solidification during the SLM causes premature destruction of the as-built sample.
(4) As-built samples are highly textured due to the layer-by-layer manufacturing which causes the sample to be anisotropic as a whole.

**Figure 7:**
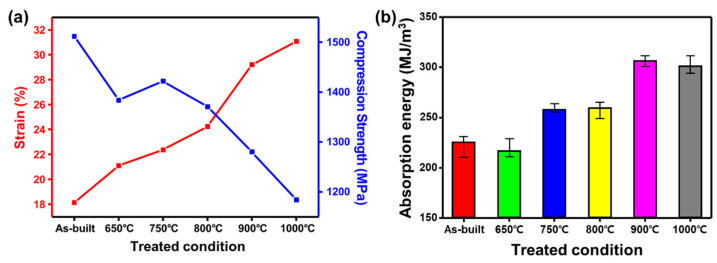
(**a**) Strain, compressive strength, and (**b**) deformation energy after fracture in different SLM Ti-6Al-4V specimens.

Therefore, the morphology of the α′-martensite, residual stresses, and texture were minimized considerably after heat treatment. Consequently, the deformability improves, but the strength of the samples drops down. The effect of heat treatment is shown by the strength and elongation values extracted from the compressive stress–strain diagrams as shown in Figure 7a.

The as-built sample has the least elongation of 21.9% and higher compressive strength (1512 MPa) which consists of a fully acicular α′-martensite phase. The work hardening of the sample is poor, and they are rapidly destroyed after yielding, resulting in very low deformability. This is further confirmed from their poor deformation; energy is also low around 213 MJ/m^3^ (Figure 7b). In the heat-treated sample at 650 °C, the elongation improves slightly, while the strength drops accordingly. The decomposition of α′-martensite is relatively smaller at 750 °C as compared to that at 650 °C, hence a slight improvement in strength. The enhancement in elongation was significant beyond 750 °C. The deformation energy increased significantly to 270 MJ/m^3^ as compared to the as-built and 650 °C conditions. From 800 °C, the elongation increases, and the strength tends to decrease considerably due to a greater relation in stress to some extent and faster decomposition of α′-martensite into lamellar α + β phase structure [21,22]. At higher heat treatment temperatures, 800 °C and 900 °C, the strength values are also dropped due to the increased lamellar structure and width of the lamellae at elevated temperatures. Lastly, at 1000 °C, the strength value was lowest (1182 MPa) with a maximum elongation (31%) and deformation energy of 284 MJ/m^3^. When the heat treatment is performed at a temperature lower than 900 °C, the strength is high, but the elongation is low. When the heat treatment is performed beyond 900 °C, the elongation is high, but the strength value is significantly reduced [21,22,23]. Previous studies show that fracture toughness of the SLM Ti-6Al-4V specimens is either smaller or similar to those of wrought alloys [24,25,26,27]. In this regard, we see that post heat treated SLM Ti-6AL-4V samples, the presence of harder and brittle α′-martensite phase in microstructure is attributed for the low absorption energy of the specimens. The enhancement in the absorption energy at higher annealing temperatures up to 900 °C could be related to the presence of transformed lamellar α + β structure, which is relatively more ductile after annealing. In addition, coarsening of the laths after annealing also enhance the absorption energy by increasing the crack tortuosity [28,29,30]. It was inferred that the microstructure evolves with relief in the residual stresses by heat and the fracture energy is improved considerably. Previous reports on tensile studies show that the tensile strength of the SLM Ti-6Al-4V alloys is 1240 ± 7.7 MPa, and after heat treatment it can change to 1068 ± 26.7 MPa. Similarly, the elongation varies from 5.79 ± 0.29% for SLM Ti-6Al-4V to 10.28 ± 0.20% after heat treatment [31]. Therefore, it can be inferred that the elongation, strength, and absorption energy can be controlled by fine-tuning of homogenized α + β lamella structure with an appropriate width, as well as the complete removal of residual stress and microstructural anisotropy due to α′-martensite or texture [32,33,34].

## 4. Conclusions

In this work, we have studied the effect of heat treatment on the microstructure and mechanical properties of SLM Ti-6Al-4V alloys. The important conclusions drawn from this study are summarized as follows:

1. The columnar grain growth in SLM Ti-6Al-4V alloy along the build direction was gradually removed after heat treatment. The decomposition of the fully α′-martensitic phase of the as-built sample produced a lamellar α + β structure gradually after heat treatment.
2. The microstructure of SLM Ti-6Al-4V alloy is almost free from columnar grains beyond 900 °C with an increased size of lamellae. The width of the lamella at 100 °C was almost more than twice the value obtained at 900 °C. The effect of heat treatment of SLM Ti-6Al-4V alloy effective for homogenizing the two-phase microstructure of the alloy.
3. The microhardness and compressive strength of Ti-6Al-4V samples were maximum in the as-built condition. The heat treatment of as-built samples contributed to an enhancement of the elongation and deformation energy at the cost of their strength.
4. The elongation (18–29%) and the absorption energy of SLM Ti-6Al-4V alloys varied from 213–284 MJ/m^3^ while the compressive strength showed a reduction from 1512 to 1182 MPa, with an increase in temperature up to 1000 °C. The optimal heat treatment condition was found to be 900 °C due to a balanced strength and ductility relationship.
5. The resultant mechanical properties of the alloy correspond to the variation in microstructural features and can be adjusted according to the heat treatment temperature. Thus, SLM a Ti-6Al-4V with a controlled microstructure through heat treatment can be ensured, as compared to those of cast products obtained through conventional manufacturing methods.

## Data Availability

The data used in this article is confidential and cannot be shared at this time.
